# Age specific aetiological agents of diarrhoea in hospitalized children aged less than five years in Dar es Salaam, Tanzania

**DOI:** 10.1186/1471-2431-11-19

**Published:** 2011-02-23

**Authors:** Sabrina J Moyo, Njolstad Gro, Mecky I Matee, Jesse Kitundu, Helge Myrmel, Haima Mylvaganam, Samuel Y Maselle, Nina Langeland

**Affiliations:** 1 Department of Microbiology and Immunology, School of Medicine, Muhimbili University of Health and Allied Sciences (MUHAS) Dar es Salaam, Tanzania; 2 Department of Paediatrics and Child Health, Muhimbili National Hospital, Dar es Salaam, Tanzania; 3 Department of Microbiology, Haukeland University Hospital, Bergen, Norway; 4 Institute of Medicine, University of Bergen, Bergen, Norway

## Abstract

**Background:**

This study aimed to determine the age-specific aetiologic agents of diarrhoea in children aged less than five years. The study also assessed the efficacy of the empiric treatment of childhood diarrhoea using Integrated Management of Childhood Illness (IMCI) guidelines.

**Methods:**

This study included 280 children aged less than 5 years, admitted with diarrhoea to any of the four major hospitals in Dar es Salaam. Bacterial pathogens were identified using conventional methods. Enzyme Linked Immunosorbent Assay (ELISA) and agglutination assay were used to detect viruses and intestinal protozoa, respectively. Antimicrobial susceptibility was determined using Kirby-Bauer disk diffusion method.

**Results:**

At least one of the searched pathogens was detected in 67.1% of the cases, and mixed infections were detected in 20.7% of cases. Overall, bacteria and viruses contributed equally accounting for 33.2% and 32.2% of all the cases, respectively, while parasites were detected in 19.2% patients. Diarrhoeagenic *Escherichia coli *(DEC) was the most common enteric pathogen, isolated in 22.9% of patients, followed by *Cryptosporidium parvum *(18.9%), rotavirus (18.1%) and norovirus (13.7%). The main cause of diarrhoea in children aged 0 to 6 months were bacteria, predominantly DEC, while viruses predominated in the 7-12 months age group. *Vibrio cholerae *was isolated mostly in children above two years. *Shigella *spp, *V. cholerae *and DEC showed moderate to high rates of resistance to erythromycin, ampicillin, chloramphenicol and tetracycline (56.2-100%). *V. cholerae *showed full susceptibility to co-trimoxazole (100%), while DEC and Shigella showed high rate of resistance to co-trimoxazole; 90.6% and 93.3% respectively. None of the bacterial pathogens isolated showed resistance to ciprofloxacin which is not recommended for use in children. Cefotaxime resistance was found only in 4.7% of the DEC.

**Conclusion:**

During the dry season, acute watery diarrhoea is the most common type of diarrhoea in children under five years in Dar es Salaam and is predominantly due to DEC, *C. parvum*, rotaviruses and noroviruses. Constant antibiotic surveillance is warranted as bacteria were highly resistant to various antimicrobial agents including co-trimoxazole and erythromycin which are currently recommended for empiric treatment of diarrhoea.

## Background

Infective diarrhoea is one of the leading cause of morbidity and mortality among children under five years in the developing world [1] and can be caused by a wide range of viruses, bacteria, or parasites [2,3]. The prevalence of the different enteric pathogens varies with the geographical area [2].

There are relatively few studies on this aspect that have been conducted in Tanzania. Most of these studies have focused on few aetiological agents of diarrhoea and were conducted in late eighties and early nineties [4-6]. There is one recent study conducted in Ifakara, Tanzania in 2004, which shows the prevalence of different aetiological agents of diarrhoea, i.e. viruses, bacteria and parasites among children with diarrhoea. In that study diarrhoeagenic *Escherichia coli *(DEC) was the predominant enteropathogen. Moreover, *Shigella *spp. and rotavirus were more prevalent in the dry season than in the rainy season and *Giardia lamblia *was more prevalent in the rainy season [7].

For quite some time children with diarrhoea in Tanzania have been and are being treated empirically with erythromycin and co-trimoxazole according to the Integrated Management of Childhood Illness (IMCI) guidelines [8]. However current information regarding antimicrobial susceptibility pattern of bacteria causing diarrhoea in children is limited and thus it is uncertain whether the recommended antibiotics are still effective.

The present study aimed at detection of enteric pathogens in children with diarrhoea to provide an update on the spectrum and age specific causes of diarrhoea. The study also determined the antimicrobial resistance pattern of bacterial pathogens.

## Methods

### Study design and settings

This cross-sectional study was conducted in Dar es Salaam, Tanzania between December 2005 and February 2006. Participants were children ≤5 years, who during the study period, were admitted due to diarrhoea at Muhimbili National Referral Hospital (MNH), Amana, Mwananyamala and Temeke Municipal Hospitals in Dar es Salaam, Tanzania. Enrolment was subjected to obtaining an informed verbal consent from parent or guardian who accompanied the child.

### Interviews

A structured questionnaire was used to obtain information of the children from the parents/guardians regarding age, sex, duration and description of the stool (watery, mucoid, or bloody) and the use of antibiotics prior to hospitalization. The definition and forms of diarrhoea were according to WHO guidelines [8].

### Weight measurements

On admission infants less than two years of age were weighed using a 25 kg Salter hanging scales (CMS Weighing equipment, High Holborn, London, United Kingdom). Children over two years were weighed on scales calibrated before each session. Weight of children was recorded to the nearest kilogram.

### Determination of nutritional status

Weight-for age Z-scores were calculated using EPI Info (USD, Inc., Stone Mountain, GA). According to WHO criteria children were considered to be undernourished if the Z-scores were less than -2SD[9].

### Collection and transportation of stool

Stool specimens were collected using wide mouthed sterile plastic containers and transported to the Microbiology laboratory at Muhimbili National Hospital within two hours of collection. A portion of specimen was stored at -20°C until further analysis at Haukeland University Hospital in Bergen, Norway.

### Isolation and identification of bacteria pathogens

Bacterial pathogens *E. coli*, *V. cholerae*, *Salmonella *spp and *Shigella *spp. were isolated and identified by conventional methods [10]. Identification of DEC was done as previously described [11].

### Antimicrobial susceptibility testing of bacterial isolates

Susceptibility testing was performed by disk diffusion method according to Clinical and Laboratory Standards Institute guidelines (CLSI) [12]. *Escherichia coli *ATCC 25922 was used as a reference control strain. Antibiotics tested were ampicillin 10 μg, amoxycillin and clavulanic acid (Augmentin) 20/10 μg, erythromycin 15 μg, ciprofloxacin 100 μg, gentamicin 10 μg, cefotaxime 30 μg, cephalothin 30 μg, co-trimoxazole 25 μg, chloramphenicol 30 μg and tetracycline 30 μg (Remel, Lenexa, USA).

According to the size of the zones of inhibition, the organisms were classified as sensitive, intermediate or resistant to a specific antibiotic according to CLSI guidelines [12]. For the purpose of this study intermediate sensitivity was considered as sensitive.

### Detection of viruses

The presence of four enteric viruses: rotavirus, norovirus, adenovirus and astrovirus were detected by commercially available enzyme linked immunosorbent assay (ELISA) according to manufacturer's instructions (Dako Ltd., Ely, United Kingdom). The tests detected specific antigens for group A rotaviruses, norovirus, adenoviruses type 40 and 41 and astroviruses, respectively.

### Detection of intestinal protozoa Cryptosporidium parvum and Giardia lamblia

The presence of *C. parvum *and *G. lamblia *antigens was tested from frozen stool samples using ImmunoCard STAT! Rapid Assay (Meridian Bioscience, Belgium). This test simultaneously detects and distinguishes between *C. parvum *and *G. lamblia *antigens in aqueous extracts of patient stool specimens.

### Ethical considerations

Ethical clearance was obtained from the Institutional Review Board of MUHAS, Dar es Salaam. Informed verbal consent was obtained from parents/guardians of the children before enrolment. Children were treated according to IMCI guidelines [8].

### Data analysis

The Statistical Package for the Social Sciences (SPSS for windows, version 10.0) was used for statistical analysis. Assuming the data follows a normal distribution, comparison of proportions and statistical significance were tested by using the Chi-square test. A p value less than 0.05 was considered statistically significant.

## Results

A total of 280 children (172 boys and 108 girls) aged 0-60 months with diarrhoea were enrolled. Of these 148(52.9%) were from Amana Hospital followed by MNH 47(16.8%), Temeke Hospital 47(16.8%) and Mwananyamala Hospital 38 (13.6%) (Table 1).

**Table 1:** Social demographic data, breastfeeding and nutritional status and type of diarrhoea of the study group

	Number (%)
**Hospital**	
MNH	47 (16.8)
Amana	148(52.9)
Temeke	47(16.8)
Mwananyamala	38(13.6)
**Sex**	
Boys	172(61.4)
Girls	108(38.6)
**Age group**	
0-6	51(18.2)
7-12	135(48.2)
13-24	67(23.9)
25-60	27(9.6)
**Antibiotic use prior to admission**	
Yes	128(45.7)
No	152(54.3)
**Exclusive B/F (0-6) months**	
Yes	6(11.8)
No	45(88.2)
**Nutritional status**	
Malnourished	79(28.2)
Normal	201(71.8)
Overall mean Zscore	-1.2453
**Type of diarrhoea**	
Acute watery	235(83.9)
Persistent	27(9.6)
Dysentery	18(6.4)

Two hundred and thirty five (83.9%) children presented mainly with acute watery followed by persistent diarrhoea in 27 (9.6%), while 28 (6.4%) had dysentery. Of the 51 children aged below six months, only six (11.8%) were exclusively breast-fed. About 97% of the children aged 7 to 12 months were still being breast-fed. Seventy nine children (28.2%) were malnourished.

At least one enteric pathogen was detected in 188 (67.1%) patients. Of these 93 (33.2%), 87 (32.2%) and 52 (19.2%) were bacteria, viruses and parasites respectively. Mixed infections with up to four different pathogens were detected in 56 (20.7%) of all cases. Most (75.9%) of the co-infections were with two pathogens, followed by three pathogens (20.4%) and a few (3.7%) were with four pathogens.

The prevalence of different enteric pathogens is shown in Table 2. DEC was the most common pathogen, isolated in 64 children (22.9%), followed by *C. parvum *detected in 51 (18.9%), rotavirus in 49 (18.1%) and norovirus in 37 (13.7%). *V. cholerae *and *Shigella spp *accounted for 5.7% and 5.4% of all cases of diarrhoea respectively. Of the DEC strains detected, 41(64.1%), 13(20.3%) and 10(15.6%) were EAEC, EPEC and ETEC respectively. All ETEC strains harboured only the stable toxin. There were no EIEC or EHEC detected in this group.

**Table 2:** Isolation frequency of enteric pathogens from under fives with diarrhoea in Dar es Salaam

	Number (%)
**Bacteria (n = 280)**	
DEC	64 (22.9%)
*V. cholerae *01	16 (5.7%)
*Shigella *spp	15(5.4%)
*S. flexneri*	10 (66.7%)
*S. dysenteriae*	5 (33.3%)
*Salmonella *spp	7 (2.5%)
*S. Typhimurium*	4 (57.1%)
*S. Enteritidis*	2 (28.6%)
*S. Typhi*	1(14.3%)
**Protozoans (n = 270)**	
*C. parvum*	51 (18.9%)
*G. lamblia*	5 (1.9%)
**Viruses **n = 270	
Rotavirus	49 (18.1%
Norovirus	37(13.7%)
Adenovirus	7 (2.6%)
Astrovirus	1 (0.4%)
**Mixed infection **(n = 270)	56 (20.7%)

All *V. cholerae *isolates were sero group OI strains of Ogawa. Of the seven *Salmonella *isolated, four were *Salmonella Typhimurium*, two were *Salmonella Enteritidis *and one was *Salmonella Typhi. Salmonella Paratyphi *was not detected. Of the fifteen *Shigella *isolates, 10 were *Shigella flexneri *and five were *Shigella dysenteriae*. *Shigella sonnei *and *Shigella boydii *were not detected.

Table 3 shows that bacteria, viruses, parasites and mixed infection were highly prevalent in young infants aged 0-6 months (25.0%-56.8%) and 7-12 months (26.1%-52.3%) (p > 0.05), the prevalence of all pathogens decreasing with increasing age. When stratifying bacterial pathogens with age groups, DEC, was significantly higher (39.2%) in the age group of 0-6 months than the rest of the age groups (p = 0.01) while *V. cholerae *was significantly higher in older children >25 months (p = 0.01).

**Table 3:** Association between risk factors and enteric pathogens isolated among under fives with diarrhoea in Dar es Salaam

Risk factors	Bacteria n (%)	Viruses n (%)	Parasites n (%)	Mixed infection n (%)
**Age groups in months** 0-6 *n = 51*	25(56.8)	21(47.7)	11(25.0)	15(34.1)
7-12 *n = 135*	40(45.5)	46(52.3)	23(26.1)	26(29.5)
13-24 *n = 67*	16(42.1)	17(44.7)	16(42.1)	11(28.9)
25-60 *n = 27*	12(66.7)	3(16.7)	2(11.1)	4(22.2)
**Sex**				
Male *n = 172*	57(48.7)	59(50.4)	29(24.8)	34(29.1)
Female *n = 108*	36(50.7)	28(39.4)	23(32.4)	22(31.0)
**Nutrition status**				
Malnourished *n = 79*	25(51.0)	22(44.9)	18(36.7)	18(36.7)
Normal nutrition status *n = 201*	68(48.9)	65(46.8)	34(24.5)	38(27.3)
**Type of diarrhoea** Acute *n = 235*	72(46.8)	75(48.7)	41(26.6)	45(29.2)
Persistent *n = 27*	12(57.1)	8(38.1)	9(42.9)	8(38.1)
Dysentery *n = 18*	9(69.2)	4(30.8)	2(15.4)	3(23.1)

Overall, 86.2% - 97.4% of all the pathogens caused acute watery diarrhoea, with the exception of *Shigella *species which accounted for 40% of cases of dysentery (p < 0.05). Thirty-five (85.4%) of the 41 children with EAEC presented with acute watery diarrhoea, while five (12.2%) and one (2.4%) presented with persistent diarrhoea and dysentery respectively. Among children harbouring EPEC, ten (76.9%) had acute watery and three (23.1%) had persistent diarrhoea. Six (60.0%), two (20.0%) and two (20.0%) of the children with ETEC had acute watery diarrhoea, persistent diarrhoea and dysentery, respectively. Among the 92 children with diarrhoea whose aetiogical agents could not be detected; 87(94.6%) and 5(5.4%) presented with acute and persistent diarrhoea respectively.

Table 4 summarizes the antimicrobial resistance pattern of different bacterial pathogens. Overall, bacterial pathogens isolated showed no resistance to ciprofloxacin and cefotaxime except for DEC which showed 4.7% resistance to cefotaxime. *Shigella *spp, *V. cholerae *and DEC showed moderate to high rates of resistance to erythromycin, ampicillin, chloramphenicol and tetracycline (56.2-100%). *V. cholerae *showed full susceptibility to co-trimoxazole (100%), while DEC and *Shigella spp *showed high rate of resistance to co-trimoxazole; 90.6% and 93.3% respectively.

**Table 4:** Antimicrobial resistance pattern of bacterial enteric pathogens isolated from under fives with diarrhoea in Dar es Salaam

Antimicrobial agent	*Salmonella spp*	*Shigella spp*	*V. cholerae*	DEC
Ampicillin	42.8	93.3	75	96.9
Augmentin	14.3	60	62.5	7.8
Erythromycin	57.1	26.7	75	90.6
Chloramphenicol	24.3	93.3	68.7	56.2
Ciprofloxacin	0	0	0	0
Co-trimoxazole	28.6	93.3	0	90.6
Tetracycline	28.5	100	93.7	71.8
Gentamicin	28.6	0	6.2	17.2
Cefotaxime	0	0	0	4.7
Cephalothin	42.9	20	62.5	31.3

## Discussion

In this study the prevalence of diarrhoea with an identified aetiology was 67.1% and consistent with previous findings from Ifakara, Tanzania [7]. Pathogens were not detected in 92(32.9%) of the children. This could be partly due to potential enteric pathogens such as helminths, *Campylobacter *spp, *Yersinia enterocolitica *and *Aeromonas *spp which were not searched for, or because of the fact that some of the children had a history of antibiotic use prior to admission.

We found bacterial pathogens in 33.3%, enteric viruses in 32.2% and the protozoas in 19.2% of patients. However when age stratification was done, the main cause of diarrohea in children aged 0-6 months were bacteria, predominantly DEC, while diarrhoea in children aged 7-12 months was more often due to viruses, mainly rotavirus and norovirus. *Vibrio cholerae *was isolated mainly in children aged above two. This age related pattern of pathogens is consistent with reports from studies conducted in other developing countries [2] and should be taken into account when considering appropriate management of childhood diarrhoea in Dar es Salaam.

Fifty six children (20.7%) had multiple infections with up to four associated pathogens, a figure comparable with findings in other developing countries [13,14]. Dual infections raise the question of whether a single pathogen is responsible for illness, or whether several pathogens act in synergy. Further studies must be performed in order to obtain a better understanding of these infections. The predominance of DEC (64.1%) among all bacterial isolates is consistent with previous reports from Tanzania and other developing countries [7,15], underlining their importance as the main bacterial causes of diarrhoea in children aged less than five years and the need to type them in order to know the types that cause diarrhoea in our setting.

Surprisingly, viruses were detected in four children with dysentery. One them had mixed infection with ETEC strain of DEC while the other three had no bacteria detected. Viruses do not normally cause bloody diarrhoea, the possible reason for this finding could be that enteric bacteria such as *Shigella spp *were not isolated because all the four children had history of taking antibiotics prior to the study or dysentery could have been caused by other enteric pathogens such *Entamoeba histolytica *which was not searched for in this study.

Overall, *Shigella *spp were detected in 5.7% of cases of diarrhoea but the frequency was significantly higher (40%) in children with bloody diarrhoea. The predominance of *S. flexneri *(66.7% of *Shigella *isolates) found in this study is consistent with the finding in Ifakara Tanzania [7].

The prevalence of *Salmonella spp *found in this study (5.7%) falls within the reported range of 1-5% of gastroenteritis cases in most developing countries [8] and the serotypes were mainly *S. Enteritidis *(28.6%) and *S. Typhimurium *(57.1%).

The high prevalence *V. cholerae *in the present study (5.7%) was due to an outbreak of cholera in the city during the time of study.

The prevalence of cryptosporidiosis reported in this study (18.9%) is higher than the 8.5% reported previously in Dar es Salaam [6]. The difference could be due to difference in HIV prevalence during the two study periods given the association between cryptosporidiosis and HIV infection [6]. However we did not screen for HIV infection in the current study. The difference could also be due to different methods used for detection of cryptosporidiosis. We found *G. lamblia *in only a minority of diarrhoea cases (1.9%), probably due to their limited role in childhood diarrhoea among children of Dar es Salaam.

We found no relationship between the enteric pathogens detected and the lack of exclusive breast feeding. This could be due to the fact that only a few children (11.2%) were exclusively breast-fed.

Our findings show that most bacterial pathogens isolated were sensitive to ciprofloxacin, cefotaxime, gentamicin, amoxicillin-clavulanic acid and cephalothin, probably due to their seldom use [16-19]. On the contrary, high rate of resistance was seen in commonly prescribed antibiotics such as ampicillin, tetracycline, including co-trimoxazole which are currently recommended for empirical treatment [8]. The high rate of resistance towards these antibiotics may be due to their overuse because they are readily available over the counter. The 4.7% prevalence of resistance of DEC to cefotaxime is noteworthy because third generation cephalosporin resistance is usually caused by expression of extended-spectrum beta-lactamase (ESBL) enzymes which may be the case in these strains although specific detection of ESBL production was not performed. ESBL producing-strains have been reported previously in septicaemic as well as Intensive Care Unit patients from Muhimbili National Hospital [20,21].

The antimicrobial resistance of *V. cholerae *observed in this study when compared with those of Urrasa *et al *[17] does show an increase in rate the of resistance to ampicillin (17% and 53.4% in 1997 and 1999 respectively versus 75% in the present study), erythromycin (18.1% and 54% in 1997 and 1999 respectively versus 75%) and tetracycline (6.45 and 41.1%% in 1997 and 1999 respectively versus 93.7%). The study also found a decrease in the rate of resistance to co-trimoxazole (96.8% and 96.6% in 1997 and 1999 respectively versus 0% in the present study). The low rate of resistance of *V. cholerae *to co-trimoxazole supports the current national policy of treating cholera with co-trimoxazole. However there is a need for continuous surveillance to monitor and track potential development of resistance.

## Conclusions

During the dry season, acute watery diarrhoea is the most common type of diarrhoea in children under five years in Dar es Salaam and is predominantly due to DEC, *C. parvum*, rotaviruses and noroviruses. High rate of resistance of bacteria to co-trimoxazole and erythromycin which are currently recommended for empiric treatment of diarrhoea and other antimicrobial agents, calls for continued antibiotic surveillance.

## Competing interests

The authors declare that they have no competing interests.

## Authors' contributions

SJM was the principal investigator, who conceived and designed study and was responsible for collection of specimens and clinical information as well as data analysis. Laboratory investigations were performed by SJM under the guidance of NG and HM. MIM, JK, HM, HM SYM and NL assisted in the development of the research proposal, data analysis and preparation of the manuscript. All authors have read and approved the final manuscript.

## Pre-publication history

The pre-publication history for this paper can be accessed here:

http://www.biomedcentral.com/1471-2431/11/19/prepub
